# Artificial Intelligence and Sensing Technologies for Vascular Access in Hemodialysis: A Narrative Review

**DOI:** 10.3390/s26196089

**Published:** 2026-09-25

**Authors:** Concetto Sessa, Elettra Lomeo, Walter Morale, Tito Gianni, Bibiana Bonerba, Giuseppe Grandaliano, Francesco Pesce

**Affiliations:** 1 Department of Nephrology and Dialysis, Maggiore “Nino Baglieri” Hospital, 97015 Modica, Italy; concetto.sessa@asp.rg.it (C.S.); walter.morale@asp.rg.it (W.M.); 2 Nephrology, Dialysis and Transplantation Unit, Department of Medical and Surgical Sciences, Fondazione Policlinico Universitario Agostino Gemelli IRCCS, 00168 Rome, Italy; elettra.lomeo@policlinicogemelli.it (E.L.); bibiana.bonerba@policlinicogemelli.it (B.B.); giuseppe.grandaliano@unicatt.it (G.G.); 3 Department of Nephrology, Sidney Kimmel Medical College at Thomas Jefferson University, Philadelphia, PA 19107, USA; txg421@jefferson.edu; 4 Department of Translational Medicine and Surgery, Università Cattolica del Sacro Cuore, 00168 Rome, Italy; 5 Division of Renal Medicine, Ospedale Isola Tiberina—Gemelli Isola, 00186 Rome, Italy

**Keywords:** hemodialysis, vascular access, arteriovenous fistula, artificial intelligence, machine learning, wearable sensors, acoustic sensing, edge computing

## Abstract

Vascular access performance determines the adequacy, safety, and cost of hemodialysis. Stenosis, thrombosis, cannulation difficulty, infection, and access-related cardiac stress remain major sources of morbidity. This narrative review examines artificial intelligence and sensing technologies for vascular access care, based on PubMed and ClinicalTrials.gov searches through 22 August 2026 with citation chaining. Existing reviews have examined artificial intelligence in dialysis broadly or prediction models in isolation. In this review, the vascular access itself is kept as the unit of analysis, and the evidence is followed across four connected stages: systemic risk stratification; prediction of maturation, dysfunction, and complications from clinical, ultrasound, and multimodal data; longitudinal monitoring with optical, acoustic, vibration, thermal, and pressure sensors; and procedural support with robotics and visualization technologies. Reported discrimination is high in retrospective, recording-level analyses, reaching areas under the curve of 0.99 and sample-level accuracy of 100%, but falls to approximately 0.71 in longitudinal designs that predict future events in the same patients; patient-level external validation and calibration are also rarely reported. No approach has yet been demonstrated to reduce thrombosis, prolong patency, avoid interventions, or lower cost relative to standard care in prospective multicenter studies. Deployment is further constrained by power budgets, duty cycling, and transmission bandwidth in wearable devices. Technical feasibility should not be read as clinical utility.

## 1. Introduction

Hemodialysis (HD) is the most widely used form of kidney replacement therapy worldwide and is life-sustaining for millions of patients with kidney failure [1,2,3,4]. During HD, blood passes through a vascular access (VA) into an extracorporeal circuit and across a semipermeable membrane, which removes excess fluid, electrolytes, and uremic solutes. The filtered blood then returns to the circulation through the VA [5]. A reliable VA is essential for adequate HD, and its performance affects clinical outcomes and quality of life [6].

Despite advances in access creation and surveillance, access type and VA complications remain important determinants of outcome. Stenosis, thrombosis, aneurysm formation, cannulation difficulty, infection, and access-related cardiovascular stress contribute to morbidity and mortality [7,8,9]. In a US National Inpatient Sample analysis covering 2005–2018, access-related complications were recorded in 1,167,886 of 9,246,553 admissions involving patients with end-stage kidney disease receiving hemodialysis (12.6%); Medicare analyses also show substantial costs associated with arteriovenous fistula (AVF) creation, maturation, and maintenance [10,11].

Current VA care relies primarily on physical examination, including inspection, palpation, and auscultation, to identify established or developing pathology. Clinicians may complement examination with dialysis-machine alarms, access-pressure and blood-flow trends, adequacy measures such as Kt/V and the urea reduction ratio, and episodic duplex ultrasound. Even with these measures, dysfunction may remain undetected until clinical signs appear. Guidelines consider the evidence insufficient to recommend routine access surveillance in addition to clinical monitoring, regard surveillance findings as supplementary, and reserve intervention for stenosis accompanied by persistent clinical indicators [12].

Advances in artificial intelligence (AI) have expanded risk stratification and clinical decision support in nephrology [13]. In VA care, models can use demographic, clinical, laboratory, dialysis-session, ultrasound, and other sensor data to predict adverse events, identify high-risk phenotypes, and tailor surveillance intensity. Wearable and point-of-care devices may also provide higher-frequency data for continuous or near-continuous assessment.

This review synthesizes recent developments in AI and sensing technologies for VA management. It covers four domains: (i) systemic risk stratification using electronic health records; (ii) prediction of VA maturation, dysfunction, and complications using clinical, ultrasound, and multimodal data; (iii) longitudinal monitoring with photoplethysmography, acoustic or vibration analysis, thermal sensing, and pressure sensors; and (iv) procedural support with robotics and visualization technologies.

The review distinguishes performance reported during model development from evidence of clinical benefit. Particular attention is given to cohort size, unit of analysis, internal versus external validation, calibration, interpretability, sensor deployment, and the clinical relevance of the reference standard. This distinction is treated as the field’s central problem rather than as a methodological caveat. Reported discrimination ranges from near-perfect values in retrospective, recording-level analyses to considerably more modest values in longitudinal designs that predict future events in the same patients. This gap is largely attributable to study design rather than to the underlying technologies, and interpreting it appropriately is necessary to determine whether any of these systems are ready for use.

Reviews published over the past two years have covered artificial intelligence in hemodialysis only broadly or have treated prediction models for arteriovenous fistula outcomes and wearable technologies in kidney failure in isolation. This review offers a single account organized around the vascular access itself, examining prediction from clinical and imaging data, longitudinal sensing, procedural technologies, and the conditions required for deployment as stages of one care pathway rather than as separate research streams. Therefore, the central question is not which model performs best in a retrospective dataset, but what must be shown before any of these systems can change how a vascular access is monitored and when it needs to be treated.

Figure 1 summarizes the four domains and links typical data sources, model tasks, and indicative translational maturity across the VA care pathway.

### Review Design, Scope, and Search Strategy

This narrative review encompasses clinical prediction studies, sensor engineering, device prototypes, and registered trials. The relevant literature is characterized by such heterogeneity in outcome definitions, units of analysis, and reference standards that a formal quantitative synthesis would risk being misleading rather than clarifying. Systematic reviews restricted to a single question, such as prediction of AVF dysfunction, have been able to apply structured appraisal tools precisely because they excluded most of the sensing and procedural literature considered here [14]. The aim of this review is complementary: to map a heterogeneous and rapidly evolving field and to delineate what the available evidence can and cannot support.

We focused on peer-reviewed research and registered clinical studies published from 1 January 2018 through 22 August 2026. Earlier sources were retained when they provided foundational clinical or technical context.

PubMed and ClinicalTrials.gov were searched on 22 August 2026. Search concepts combined hemodialysis or haemodialysis with vascular access, arteriovenous fistula, arteriovenous graft, stenosis, thrombosis, maturation, failure, machine learning, artificial intelligence, prediction, ultrasound, photoplethysmography, acoustic, bruit, vibration, thrill, accelerometer, thermal sensor, pressure sensor, wearable, robotics, near-infrared visualization, augmented reality, or virtual reality. Reference lists and citing articles of relevant reviews and primary studies were also screened. Supplementary Table S2 reports the complete Boolean strings, with field tags and the number of records retrieved for each, so that the search can be reproduced, critiqued, or extended to other databases.

English-language records were included when they reported model development or validation, sensor or device evaluation, a clinically oriented monitoring prototype, a registered study, or an evidence synthesis directly relevant to VA care. Studies of general dialysis monitoring or non-HD vascular access were retained only when they provided clearly labeled indirect evidence. Coverage was cross-checked against recent dedicated evidence syntheses [15,16,17,18,19,20].

Titles, abstracts, registry records, and accessible full texts were screened by one reviewer. Relevant records were grouped by topic, and reference lists and citing articles were used for citation chaining. Because this review was not designed as a systematic review, we did not use a PRISMA selection process, perform independent duplicate screening, conduct a formal risk-of-bias assessment, or perform a meta-analysis. Restricting the search to PubMed and ClinicalTrials.gov may have resulted in the omission of specific engineering conference proceedings indexed in IEEE Xplore and records unique to Embase, Scopus, or Web of Science, particularly sensor prototypes reported outside the clinical literature. Readers should therefore consider the coverage of the hardware sections as indicative rather than exhaustive. These limitations are revisited in Section 7.

Three reviews published while this article was in preparation partially overlap with its scope and therefore warrant explicit comparison. A systematic review of machine learning for AVF dysfunction searched four databases and applied PROBAST to ten studies, concluding that tree-based ensembles discriminate well but that external and prospective validation remain largely absent [14]. A narrative review of artificial intelligence in hemodialysis addressed five domains, including vascular access management, as well as hemodynamic and volume management, dialysis adequacy, and anemia [21]. A third review surveyed artificial intelligence and machine learning across dialysis care and identified vascular access monitoring as one of five application areas, with image-based analysis reaching an area under the receiver operating characteristic curve (AUC) of approximately 0.96 [22]. The first review is narrower in scope compared to the present work and does not address sensing hardware, procedural support, or implementation. The other two reviews are broader but treat vascular access as one topic among several, without examining acquisition conditions, units of analysis, or device-level constraints. The present article instead maintains the vascular access as the unit of analysis throughout and follows the evidence from model development through the actual requirements of deployment.

During the preparation of this manuscript, the authors used ChatGPT, GPT-5.6 Pro (OpenAI, San Francisco, CA, USA) for language editing, reference cross-checking, document formatting, and preparation of the schematic in Figure 1. The authors reviewed and edited the output and take full responsibility for the content of this publication.

## 2. Systemic Risk Models and Their Relevance to Vascular Access

Systemic outcomes influence VA planning and monitoring. Access type is associated with survival, and intradialytic hemodynamic instability is associated with access thrombosis, although these associations require careful adjustment for confounding [23,24,25]. This section is deliberately limited to systemic models whose predictors or outcomes are directly related to the vascular access. General hemodialysis prediction has been reviewed elsewhere and is therefore not discussed here [21,22].

Machine-learning models for mortality in patients receiving HD report AUCs of approximately 0.89 to 0.90 in retrospective cohorts, and one externally validated extreme gradient boosting (XGBoost) model achieved an AUC of 0.892 with a Brier score of 0.158 [26,27]. Of direct relevance, an elastic-net penalized Cox model identified the initial central venous catheter as one of the variables associated with higher mortality in male patients with diabetes receiving HD, again highlighting the clinical association between access type and survival in modeled form [28].

Chaudhuri et al. used XGBoost with nearly 300 variables to identify patients at high risk of multiple hospitalizations within 1 year across Dialysis Hospitalization Reduction Program clinics, where individualized interdisciplinary plans were subsequently developed for these patients. Program clinics had fewer hospitalizations and in-hospital days compared to matched control clinics (mean differences, −0.155 ± 0.38 and −0.97 ± 2.78 per patient-year, respectively) [29]. These clinic-level reductions require prospective confirmation before they can be attributed to the prediction model itself.

Intradialytic hypotension is relevant to vascular access because recurrent episodes of hypoperfusion may compromise access patency. In a systematic review of 38 models across 16 studies, artificial neural networks and XGBoost reported the highest AUCs (0.98 and 0.97); however, neither of the two highest-performing studies reported external validation or calibration, and 14 studies carried a high risk of bias [30]. This pattern recurs throughout this review: the strongest reported performances come from studies with the most limited validation.

Frailty and cognitive impairment have been linked to treatment adherence and VA thrombosis [31,32,33]. A stacking model using 12 predictors, including VA type, identified older patients at risk of cognitive frailty, with an AUC of 0.832 on external validation in 269 patients [34]. Hsieh et al. combined Internet of Medical Things data with electronic medical records to predict complications, including hypotension and AVF deterioration or obstruction [35]. The single-center, internally split design illustrates the potential of multimodal connected-care data but does not establish transportability.

None of these models incorporated causal structuring or propensity-based adjustment to distinguish the contribution of the VA from the baseline characteristics that influence the VA type a patient receives in the first place. Where matching was used, it was applied at the dialysis-clinic level rather than at the patient level [29], which balances facility case mix but leaves individual-level confounding intact. This distinction is relevant because catheter use, frailty, and prognosis are all closely interrelated [7,36]: a model that predicts mortality more accurately in patients dialyzing through a catheter has not, by itself, learned anything about the catheter. Until directed acyclic graphs, propensity score methods, or comparable approaches are applied to these cohorts, systemic models should be considered tools for prioritizing follow-up rather than evidence for access decisions.

Overfitting occurs when a model learns idiosyncrasies of the development data rather than reproducible predictive patterns [37]; inadequate sample size and complex feature sets amplify this risk and destabilize variable selection [38,39]. Future studies should define the intended clinical use in advance, split the data at the patient level, report calibration and decision-curve analyses, and evaluate model performance prospectively across populations and centers.

## 3. AI for Vascular Access Prognosis and Surveillance

Prediction models have addressed AVF maturation, functional use, recurrent intervention, thrombosis, stenosis, high flow, and aneurysm phenotype. Meng and Ho reviewed 14 studies, and Yao et al. reviewed 11; more recent systematic reviews and meta-analyses reached similar conclusions about high or unclear risk of bias, inconsistent outcomes, sparse calibration, and limited external validation [16,17,18,19].

A large European study developed the AVF failure model (AVF-FM) using 46 routinely collected variables from more than 13,000 patients in the European Clinical Database [40,41]. XGBoost achieved an AUC of 0.80 (95% CI 0.79–0.81) with good reported calibration. Leading predictors included prior AVF complications, dynamic arterial and venous pressures, recirculation, and blood-pump flow; many inputs were captured automatically by dialysis-machine sensors. External validation was not reported [40].

Ada et al. compared four models for 1-year AVF failure in a single-center cohort. XGBoost achieved the best mean cross-validated AUC (0.950). The least absolute shrinkage and selection operator (LASSO) model achieved a mean cross-validated AUC of 0.934 and was translated into a six-variable bedside score using albumin, body mass index, age, urea, creatinine, and smoking status [42]. Independent clinical studies also support associations of albumin, age, body mass index, and smoking with AVF outcomes [43,44,45,46]. Urea may reflect uremic burden, whereas creatinine is influenced by muscle mass [47,48].

Preoperative vessel mapping and early postoperative assessment are attractive targets. An early study used an artificial neural network to predict radiocephalic AVF maturation [49].

Hofmann et al. used LASSO and random forest (RF) classifiers in 73,031 patients to predict conversion back to catheter use within 1 year after AVF creation. AUCs ranged from 0.541 to 0.571, suggesting limited discrimination and that demographic and clinical variables alone may not support accurate individual prediction [50].

Doneda et al. compared machine-learning models using preoperative data from 156 patients at multiple European centers. k-nearest neighbors achieved a reported accuracy of 96.8% [51]. The unit of analysis and validation scheme should be considered when comparing this result with independent patient-level cohorts.

Cho et al. used staged feature selection and XGBoost to predict AVF maturation from demographic and preoperative ultrasound data in 492 patients. Mid-forearm cephalic-vein diameter was the leading predictor (AUC 0.869; F1 score 0.883), and a 4.5 mm cutoff was associated with 89.5% maturation [52].

Li et al. trained XGBoost in 59,674 patients from the Vascular Quality Initiative to predict successful VA use at 1 year. Preoperative anatomic features contributed most, and the authors released code for point-of-care use [53].

The PREDICT-AVF study pooled 914 patients from 39 centers to predict recurrent interventions after radiocephalic AVF creation. A baseline RF model achieved a 1-year AUC of 0.75, whereas Cox models using 4–6-week or 12-week ultrasound alone achieved AUCs of 0.77 and 0.76, respectively. The authors deployed a web application, but validation outside the trial network is still needed [54].

Imaging-AI studies are smaller and more exploratory. Poushpas et al. applied tensor decomposition and recurrent classification to 61 B-mode ultrasound cine loops; the best cropped-video model achieved an AUC of approximately 0.82 [55]. Chien et al. combined clinical, flow, and angiographic data in 28 patients after angioplasty. An artificial neural network predicted long-term fistula function with an AUC of 0.75 [56]. These studies support multimodal feasibility but are not sufficient for clinical deployment.

For AVF thrombosis, Shu et al. developed a model in 974 patients. RF performed best (AUC 0.984), and Shapley additive explanations identified the number of prior surgical interventions, stenosis, free fatty acids, platelet count, and C-reactive protein as leading predictors [57]. Zhang et al. compared logistic regression with XGBoost for AVF thrombosis. In validation, logistic regression had a slightly higher AUC (0.955 vs. 0.954), and the authors proposed it as an interpretable screening baseline [58]. Neither thrombosis study used a multicenter prospective design or external validation. The selected predictors are clinically plausible and consistent with literature linking platelet indices, inflammation, intradialytic hypotension, and anemia to access failure or thrombosis [59,60,61,62].

Shu et al. also compared models for AVF stenosis in adults receiving HD. XGBoost provided the best discrimination. Shapley analysis identified the number of surgeries, duration of AVF use, prothrombin time activity, lymphocyte count, triglycerides, vitamin B12, and C-reactive protein as leading predictors [63].

A bootstrap forest model using age, first AVF blood flow, diabetes, and dyslipidemia predicted high-flow AVF with 86% sensitivity and 83% specificity; the study did not establish that model-guided care improved cardiac outcomes [64].

Zhang et al. developed a convolutional neural network (CNN) that classified arteriovenous access aneurysms as advanced or not advanced from digital photographs, reporting an AUC of 0.96 in a validation set of 402 images [65].

Across prognosis studies, reported discrimination is often high, but comparisons are limited by heterogeneous outcomes, selective reporting, class imbalance, and unclear separation between patients, visits, images, recordings, and augmented samples. Performance estimates should therefore be reported at the patient level whenever the intended use is patient-level decision support.

## 4. Sensor Technologies for Longitudinal Vascular Access Monitoring

VA pathology can progress between in-center assessments. General experience with ambulatory cardiac wearables provides indirect technical context for repeated physiologic signal acquisition [66]. VA-specific wearable and point-of-care sensors can repeatedly measure optical pulse signals, bruit, thrill, temperature, pressure, or wall motion and may complement physical examination and duplex ultrasound [15,67].

The principal modalities are optical photoplethysmography, acoustic or vibration sensing, and soft thermal or pressure sensors. Their evidence should be interpreted according to the number of independent patients, the reference standard, and whether validation separated patients rather than signal segments.

### 4.1. Optical Sensing and Photoplethysmography

Photoplethysmography (PPG) estimates pulsatile blood-volume changes from optical absorption and can be implemented in wearable devices [68]. Studies have combined PPG features with machine learning to classify VA quality, patency, flow limitation, or stenosis. Reported accuracy reached 92.05% in one study [69], and several platforms have been designed specifically for HD access monitoring [69,70,71].

Julkaew et al. developed DeepVAQ, a convolutional neural network for classifying VA quality from PPG signals. It achieved 92.13% accuracy and an AUC of 0.8605, outperforming decision-tree and k-nearest-neighbor classifiers [72].

Using a dual optical PPG sensor, Du and Stephanus applied a Levenberg–Marquardt neural network to classify AVF stenosis in 11 patients, reporting 94.8% accuracy. Because repeated optical samples rather than 11 independent patient outcomes were analyzed, the result should not be interpreted as patient-level accuracy [73].

Chiorboli et al. developed a phantom that reproduced VA anatomy, mechanics, and hemodynamics for controlled PPG testing. It generated flows of 400–700 mL/min with less than 1% error and waveforms comparable to in vivo signals. This platform may support standardized preclinical validation [74].

Optical systems remain sensitive to probe placement, skin contact, ambient light, motion artifact, access depth, and differences in access configuration. Prospective studies should therefore report placement protocols and patient-level repeatability in addition to classification performance.

### 4.2. Acoustic and Vibration-Based Monitoring

VA bruit and thrill encode flow and turbulence characteristics that may change with stenosis. Foundational studies applied deep learning directly to AVF bruit recordings and combined empirical mode decomposition with one-dimensional convolutional neural networks [75,76]. Song et al. later combined time-frequency and signal-complexity features with neural networks to detect AVF stenosis [77].

A pilot study using a new-generation digital stethoscope found that sound-derived power and frequency variables distinguished stenotic from nonstenotic AVFs. The authors emphasized the need for standardized acquisition [78]. Chang et al. found that loudness-based acoustic features normalized after angioplasty in arteriovenous fistulas, with no comparable discrimination between functional and dysfunctional arteriovenous grafts [79].

Ion Titapiccolo et al. trained a deep-learning model to identify high-pitched bruits from digital-stethoscope recordings in 65 patients at 12 centers. The model achieved an AUC of 0.94, sensitivity of 97.1%, and specificity of 73.8% [80].

Liu et al. fused blood-flow sounds from five consecutive AVF locations and reported up to 93.9% accuracy for anastomotic stenosis detection [81]. Zhang et al. trained a convolutional-block-attention and Long Short-Term Memory network on 800 patients and reported an AUC of 0.99 and precision of 99% for binary classification of normal versus stenotic fistula sounds [82]. For edge deployment, Wang et al. developed a 2.37 MB lightweight CNN and reported 100% sample-level accuracy from 3819 spectrograms derived from 119 patients; external patient-level validation was not reported [83].

Zhou et al. trained a vision transformer using 2565 AVF sound recordings from 433 patients and reported sensitivity of 92.4% [84]. Park et al. trained ResNet50 models on mel spectrograms from 40 patients and reported an AUC of 0.99 for stenosis requiring angioplasty, with gradient-weighted class-activation maps used as visual explanations [85].

Chen et al. used pulse-radar sensing and support-vector-machine classification in 46 patients to monitor vascular-wall motion and detect AVF flow dysfunction. The classifier achieved 100% specificity at detection thresholds of 500 to 750 mL/min and a maximum sensitivity of 95.2% at a threshold of 750 mL/min [86]. Lin et al. used contrastive learning with 557 unlabeled recordings drawn from a corpus of 27,406 documented dialysis-session blood-flow sounds to train a convolutional neural network for arteriovenous graft stenosis, reporting 92.8% accuracy [87].

Longitudinal and prospective designs provide more clinically relevant tests. Chung et al. analyzed 437 weekly recordings from 84 patients to predict dysfunction requiring intervention within 10 days; the separate test-set AUC was 0.7112 [88]. A prospective matched study evaluated the Hemodialysis Vascular Sound Index in 202 patients and reported AUCs of 0.90–0.94 for reduced-flow thresholds, while cautioning that matched sampling may overestimate real-world screening performance [89]. A prospective pilot of the HAVSE surface accelerometer studied 19 patients before and after angioplasty [90].

A 2026 wireless thrill sensor combined dual accelerometers with motion-artifact cancellation and was evaluated in benchtop systems, canine models, and nine patients. The device measured spatial and temporal thrill signatures associated with flow and lesion location, but the human cohort was exploratory. Motion-artifact cancellation in this device relied on a differential pair of accelerometers, a high-resolution unit placed over the VA, and an auxiliary unit measuring limb motion, with a reported 36.6 dB improvement in signal-to-noise ratio during voluntary arm movement; the analysis band was restricted to 100–200 Hz by a second-order Butterworth filter. Robustness was assessed against limb motion, posture, speech, walking, skin temperature between 36 and 46 °C, and sweating. Patient data acquisition consisted of short recordings obtained outside the dialysis session [91]. Performance under the mechanical vibration generated by the blood pump during treatment was therefore not established. The chosen passband lay above the fundamental rotation frequency of a roller pump, thus providing some degree of implicit rejection; however, implicit rejection is not measured rejection, and intradialytic use of accelerometric sensing should not be assumed from these data. The same consideration applies to any acoustic or vibration system intended for acquisition during treatment rather than between sessions, and this is a reporting gap the field should close.

Registered studies illustrate the early translational stage. AVF-MONITOR (NCT07318025) is a not-yet-recruiting, single-center proof-of-concept study with a target enrollment of six participants [92]. Dialysis Access Monitoring Using a Digital Stethoscope-Based Deep Learning System (NCT05105503) is completed and enrolled 22 participants [93]. Pan also described an AI and Internet of Things home-monitoring system that combined audio sensing, convolutional neural networks, and on-device (edge) inference [94].

Acoustic and vibration studies are affected by probe pressure and position, background noise, motion, access geometry, and repeated recordings from the same patient. Validation should therefore use patient-level data separation and report performance across devices, centers, and access types.

### 4.3. Soft, Thermal, Pressure, and Implantable Sensors

A soft thermal sensor based on thermal anemometry has been developed to estimate VA blood flow noninvasively and detect flow reductions in real time in benchtop and large-animal models. The authors propose wireless alerting, but it has not been implemented [95]. A systematic review of eight studies of wearables in HD reported monitoring of physical activity, electrolyte imbalance, fluid status, and oxygen saturation; no included study addressed vascular access [96].

Bateman et al. created a wireless, battery-free arteriovenous graft with an embedded capacitive pressure sensor and inductive coupling. In phantom models, the device detected arterial and venous stenoses, but clinical implantation has not yet been demonstrated [97]. Luo et al. developed a flexible capacitive sensor that captured wrist pulse signals in healthy volunteers and AVF tremor signals in patients receiving dialysis [98].

Soft and implantable systems must also address biocompatibility, long-term signal stability, sterilization, power or interrogation requirements, manufacturing reproducibility, cybersecurity, and the clinical pathway for responding to alerts.

Taken together, sensor studies show a progression from benchtop validation to small clinical cohorts, but few have demonstrated prospective clinical effectiveness or improved access outcomes.

Table 1 summarizes representative approaches by domain, data modality, model task, representative example, and development stage.

### 4.4. Deployment Constraints: Power, Duty Cycling, and Data Transmission

The main limitation to continuous VA monitoring is energy, not algorithmic performance. The most refined device reported in the recent literature, a wireless thrill sensor built around a high-resolution accelerometer pair, is powered by a 90 mAh lithium-ion cell. Sampling at 1600 Hz with Bluetooth Low Energy transmission, it supports approximately 50 min of continuous monitoring and wireless streaming [91]. A system described as continuous operates, in practice, as a duty-cycled one, and its published use consists of short acquisitions before or between dialysis sessions rather than uninterrupted wear. This is not a criticism of the device, which is state of the art, but it illustrates the design constraints that every wearable in this field currently faces.

Transmission imposes a second, independent constraint. Raw acoustic monitoring at a sampling rate of 8 kHz with 16-bit resolution generates a stream of approximately 128 kbit/s; a mel spectrogram computed from it is smaller but still substantial when produced continuously. Low-power wide-area networks cannot support this: data rates fall as the spreading factor increases, reaching as low as a few kbit/s at best in the sub-GHz bands. Furthermore, European regulations restrict end devices in the 868 MHz band to a 1% duty cycle, equivalent to approximately 36 s of transmission per hour per sub-band [99]. This mismatch is one to two orders of magnitude in throughput before the duty-cycle limit is even applied, so streaming raw audio or full spectrograms over such a link is not a matter of optimization but of arithmetic. Bluetooth Low Energy transmission, with a physical-layer rate of 1 to 2 Mbit/s, comfortably carries short bursts and is what current prototypes use [91,94], at the cost of requiring a nearby gateway and drawing appreciably more current while the radio is active.

These two constraints converge on a single architecture. Feature extraction and inference must occur on the device, and only compact descriptors, a classification, or a trend value should be transmitted. This has already been proven to be feasible. A lightweight convolutional neural network of 2.37 MB has been trained for VA stenosis classification from blood-flow sounds with deployment on constrained hardware as an explicit design goal [83]. Also, an Internet of Things home-monitoring system combining audio sensing with on-device inference has been described for AVF surveillance [94]. On-device inference also reduces the transfer of identifiable physiological recordings, which benefits data protection rather than bandwidth alone.

Duty-cycle design should follow disease dynamics rather than battery convenience. Stenosis in an arteriovenous access typically progresses over weeks; therefore, a schedule of short recordings, on the order of tens of seconds once or twice daily or at each dialysis session, may be sufficient to reconstruct a trajectory. This approach is consistent with the longitudinal designs that have produced the most clinically meaningful results so far [88,89]. In contrast, acute thrombosis is the clinical event that could justify continuous sensing, and it is precisely the event no current device can monitor for, given that no reported platform can sustain uninterrupted acquisition beyond approximately one hour without recharging. Thus, claims that a wearable enables early detection of VA thrombosis should be accompanied by a description of the power budget required to make such detection possible.

Reporting in this field has not yet fully addressed these considerations. While sensor studies routinely report classification metrics, they rarely report the sampling rate alongside the fraction of time the device is active, energy consumed per inference, expected operating time on a single charge, recharge or battery-replacement interval, and transmitted payload size. These parameters determine whether a system can be worn by a patient receiving dialysis three times weekly, just as much as discrimination determines whether the system can detect the target condition at all, and we suggest reporting them alongside performance as part of routine device characterization: a device that requires twice-daily recharging, even with an AUC of 0.95, may have limited utility in clinical practice.

## 5. Procedural Support, Simulation, and Visualization

AI-based systems are being developed for procedural workflows, including robotic needle guidance and automated imaging. An AI-enabled ultrasound-guided handheld robot has been tested for femoral vascular access, providing indirect evidence for robotic needle guidance rather than direct evidence of HD cannulation [100].

Thompson et al. conducted a first-in-human feasibility study of machine-learning-trained, ultrasound-guided, power-assisted needle insertion for HD cannulation. First-attempt cannulation was successful in six out of seven procedures, with no reported complications; the sample was too small to establish comparative safety or effectiveness [101].

Carroll et al. developed robotic tomographic ultrasound, a semiautonomous system that acquires three-dimensional B-mode and Doppler data and uses cloud-based software for preoperative mapping, maturation assessment, and monitoring. The system has so far been reported only as a prototype, with simulation and optimization data and no clinical validation against duplex ultrasound; prospective evaluation of accuracy, operator burden, and downstream outcomes is therefore still needed [102].

Near-infrared vein visualization is distinct from augmented reality. A meta-analysis in children and adolescents found that near-infrared devices could reduce attempts or procedure time in some peripheral intravenous cannulation settings [103]. However, this evidence is indirect and should not be extrapolated to HD AVF cannulation without dedicated studies. We identified no clinical study of augmented-reality-guided HD access cannulation.

Virtual reality (VR) has been evaluated mainly as a distraction or rehabilitation intervention rather than as an AI-enabled access technology. A systematic review and meta-analysis of 12 randomized trials in patients receiving dialysis reported improvements in depression, anxiety, social functioning, self-efficacy, and 6 min walk distance, with no significant effect on gait speed, balance, sit-to-stand performance, timed up-and-go, or fatigue [104]. In a multicenter pilot involving 148 patients, immersive VR was associated with improved well-being and small changes in blood pressure and heart rate [105].

In a pilot study, Rodríguez de Galvis et al. reported a small but significant reduction in cannulation pain, no significant change in anxiety, and mixed patient acceptance [106]. A network meta-analysis also identified visual distraction among interventions associated with lower AVF cannulation pain [107]. These findings support further controlled studies of procedural comfort; they do not demonstrate improved VA outcomes or quality of life from routine VR use.

Agent-based simulation has also been used to examine strategies to improve VA creation, illustrating how digital models may test system-level pathways before implementation [108].

Procedural systems should be evaluated against standard practice for first-attempt success, complications, procedure time, operator learning, patient experience, and resource use. Feasibility alone is insufficient to establish clinical benefit.

## 6. Implementation, Health Economics, and Policy Context

Implementation requires attention to workflow fit, clinical responsibility, privacy, cybersecurity, maintenance, and cost. Medicare analyses and a single-center facility-level analysis show that access creation, catheter use, and repeated interventions affect expenditure [11,109], but neither establishes the cost-effectiveness of AI monitoring. A modeling study compared endovascular and surgical AVF creation using US Medicare fee-schedule costs and sensitivity analyses across cost, maturation, patency, and reintervention inputs [110].

Formal economic evaluations of AI-based surveillance should compare the full pathway, including device acquisition, connectivity, training, false-positive referrals, imaging, procedures, hospital use, access longevity, and patient time. Any projected savings from earlier detection or fewer interventions remain hypothetical until evaluated prospectively.

Tools that use data already captured by dialysis machines may impose less documentation burden than systems requiring manual entry. Home or wearable monitoring adds requirements for reliable placement, charging or battery replacement, connectivity, patient training, troubleshooting, and a defined clinical team responsible for alerts. An alert without an agreed response pathway may increase workload without improving outcomes. In practice, a surveillance system that flags a stenosis should report its confidence, and that confidence should be calibrated rather than asserted. Distribution-free methods such as conformal prediction generate prediction sets with finite-sample coverage guarantees and enable a model to abstain when evidence is insufficient [111]; this converts an implicit decision threshold into an explicit operating point that a clinical team can inspect and renegotiate. Without such calibration, the false-positive burden of a device that acquires signals daily falls on the same staff who must act on each alert, making alarm fatigue the predictable outcome. Governance should therefore specify, before deployment, who receives an alert, within what time interval it must be reviewed, what examination or imaging it triggers, and how frequently the operating point must be re-evaluated against the observed positive predictive value in that unit. Implementation experience with artificial intelligence in nephrology and related specialties suggests that such arrangements persist when institutionalized as recurring interdisciplinary routines with documented decisions and instead break down when left to individual judgment [112].

## 7. Methodological Limitations and Future Directions

The main barrier to translation is not a lack of candidate algorithms but a lack of evidence that performance is reproducible and clinically useful. Many studies are retrospective, single-center, or based on a small number of patients with many repeated signals. Random splitting of images or recordings from the same patient can leak patient-specific information into the test set and inflate performance.

Dataset shift can arise from differences in patient populations, access type and location, devices, sensor placement, dialysis protocols, referral thresholds, and disease prevalence. Ground truth is also imperfect: angiographic stenosis, ultrasound flow, clinician examination, intervention, and subsequent access failure measure related but nonidentical constructs. A model can therefore appear accurate against one label while being poorly aligned with the clinical decision it is intended to support.

Future studies should prespecify patient-level training, validation, and test sets and justify sample size. They should report missing-data handling, calibration, confidence intervals, and subgroup performance and evaluate decision thresholds against a clinically meaningful comparator. Prospective external validation should span centers, devices, and access configurations. Pragmatic trials should measure intervention timing, access patency, catheter exposure, hospitalization, patient-reported experience, and cost.

Multicenter validation is the single most consistent gap in the literature, and the obstacle is rarely a lack of scientific interest but rather data governance. Federated approaches, in which model updates rather than patient-level recordings cross institutional boundaries, have been implemented in nephrology precisely to reconcile local data sovereignty with collective learning and have been described alongside structured interdisciplinary review of model outputs as a way to keep clinicians inside the development loop [112]. For vascular access, where almost every published dataset is single-center and acquisition protocols differ between units, this offers a realistic route to the external validation that the field has been unable to produce by other means. It also forces the standardization of variable definitions and acquisition conditions as a precondition rather than an afterthought.

Multimodal fusion may combine PPG, bruit or thrill, dialysis-machine parameters, clinical history, and ultrasound, but additional modalities should be included only when they improve transportable performance or clinical utility. Transparent uncertainty estimates and interpretable summaries are particularly important when outputs trigger referral or intervention. Methodological innovation should be coupled with careful trial design and reporting [113].

Edge or on-device inference may reduce latency and the transfer of identifiable data, provided that power use, failure modes, software updates, and cybersecurity are addressed in the system design. Connected monitoring should be evaluated as a complete care pathway rather than an isolated algorithm or sensor.

A cautionary precedent is available from a more mature application of the same technology. In population-based randomized evaluation of AI-supported mammography screening, a substantial increase in cancer detection was accompanied by a considerably smaller reduction in interval cancers [114]. Correspondence on that trial argued that increased detection must not be conflated with improved patient outcomes, and that evidence of reduced advanced-stage disease and ultimately of mortality benefit remains necessary before routine implementation [115]. The same exchange raised concerns about automation bias, noting that AI prompts can shift experienced readers’ decisions and that recalls attributable to the algorithm should be quantified rather than assumed neutral [116,117]. VA surveillance is at a far earlier stage and has no comparable trial. This is what makes the analogy instructive rather than reassuring: if the question of clinical benefit remains contested in a setting with randomized population-level data, it cannot be regarded as settled here on the basis of retrospective discrimination alone.

This review has several limitations. It was non-systematic, focused on English-language records identified through PubMed, ClinicalTrials.gov, and citation chaining, did not reproduce subscription-database export workflows, and did not perform a formal risk-of-bias assessment or meta-analysis. Rapid publication in this field also means that registry status and online-first metadata may change.

## 8. Conclusions

No artificial intelligence or sensor-based approach to hemodialysis vascular access currently relies on prospective, externally validated evidence sufficient to demonstrate superior patient-level outcomes, and none can be recommended for routine clinical deployment on the data available today. This claim concerns the evidence base, not the technologies themselves, and the distinction matters.

Technical feasibility is no longer the limiting factor. Acoustic, vibration, optical, thermal, and pressure sensing all capture signals that track access flow and change in the expected direction after angioplasty, and models built on clinical, laboratory, and ultrasound data discriminate well within the cohorts that produced them. What currently remains unclear is whether acting on those signals leads to any noticeable changes for patients. The literature reports no reduction in thrombosis, no prolongation of patency, no decrease in interventions or hospitalizations, and no cost-effectiveness advantage over standard care, because the studies capable of demonstrating such effects have not been performed.

The gap between the two is visible in the numbers themselves. Retrospective analyses that split recordings rather than patients report AUCs approaching unity and, in one case, perfect sample-level accuracy. Conversely, the longitudinal study that predicted an intervention within ten days in the same patients reported an AUC of 0.7112. The second figure is the more informative, and it is the one against which future work should be judged.

What would settle the issue is not a better architecture but rather a prospective multicenter study with patient-level separation of training and test data, a prespecified and clinically meaningful reference standard, calibrated outputs with an explicit operating point, a defined response pathway for positive findings, and outcomes measured as intervention timing, access patency, catheter exposure, hospitalization, patient experience, and cost. Device studies should additionally report the power budget, duty cycle, and transmission payload, which are critical determinants of whether the system can be worn at all. Until such evidence exists, these technologies should be described as promising and considered investigational, and the distinction between what is technically possible and what is clinically useful should be maintained in the language used to report them.

## Figures and Tables

**Figure 1:**
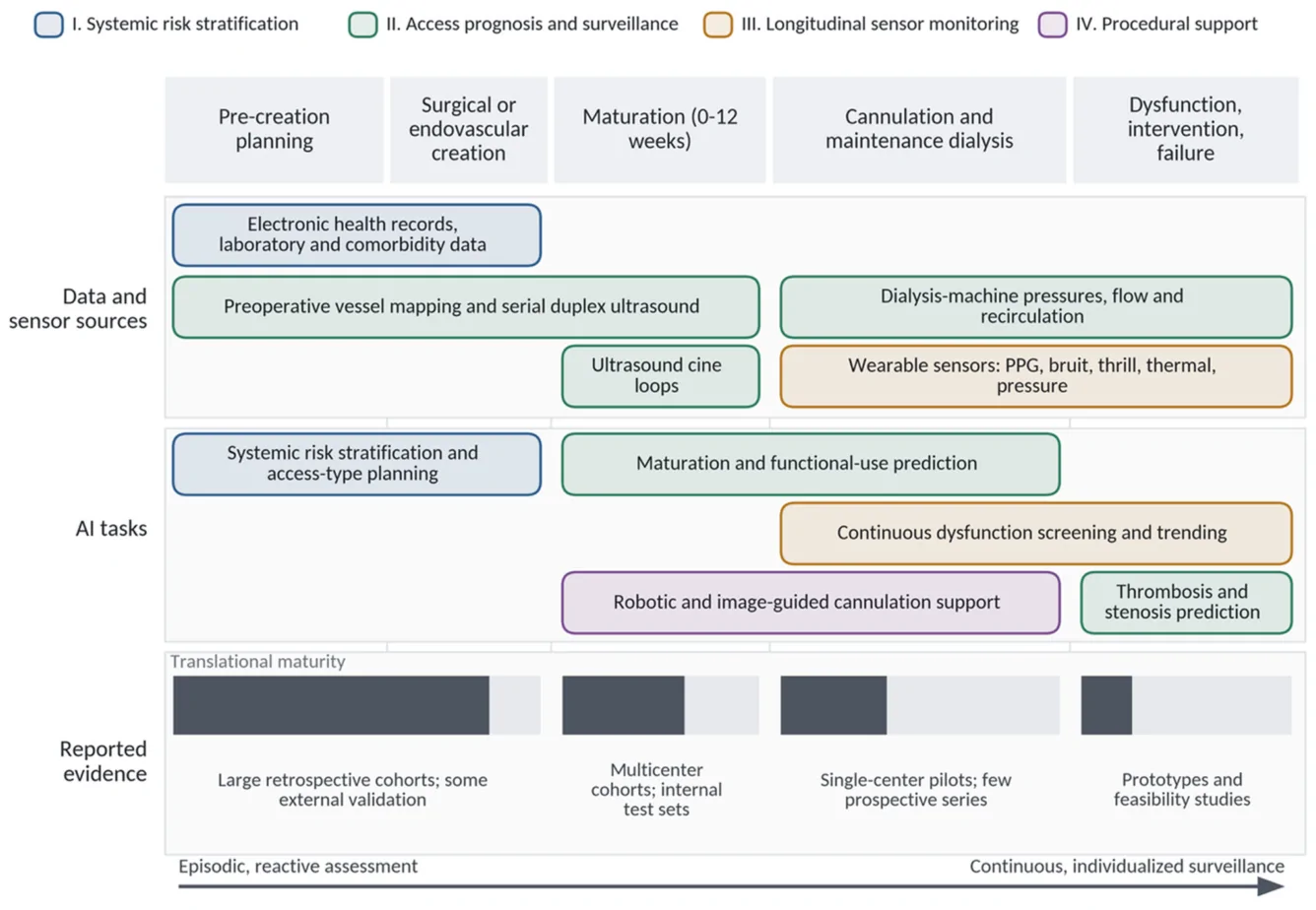
Artificial intelligence and sensing technologies for hemodialysis (HD) vascular access (VA) management. The framework groups applications into four domains: (I) systemic risk stratification; (II) VA prognosis and surveillance; (III) longitudinal sensor-based monitoring; and (IV) procedural support. Each domain is linked to representative data sources, AI tasks, and an indicative stage of technological maturity. AI, artificial intelligence; EHR, electronic health record; PPG, photoplethysmography.

**Table 1:** Representative artificial intelligence and sensor approaches for hemodialysis vascular access management, with study design and evidence of clinical benefit.

Domain and Study	Access Type	Independent Patients (n)	Data or Sensor Modality	Clinical Target	Design, Data Separation, External Validation	Discrimination	Evidence of Improved Clinical Outcome
Systemic risk, XGBoost mortality model [27]	Mixed	NR	EHR, laboratory, session data	Mortality at 1, 4, 7 years	Retrospective; external validation reported	AUC 0.892; Brier 0.158	None
Hospitalization risk, DHRP program [29]	Mixed	Clinic-level cohort	EHR, ~300 variables	Multiple hospitalizations within 1 year	Retrospective; matched control clinics (clinic level, not patient level)	NR	Fewer hospitalizations at clinic level; not attributable to the model
AVF prognosis, AVF-FM [40]	AVF	>13,000	EHR and dialysis-machine sensor data	AVF failure	Retrospective; internal; no external validation	AUC 0.80 (95% CI 0.79–0.81); calibration reported	None
AVF prognosis, PREDICT-AVF [54]	Radiocephalic AVF	914, 39 centers	Clinical and ultrasound	Recurrent intervention	Prospective cohort; internal test set; no validation outside trial network	1-year AUC 0.75; ultrasound-only Cox 0.77 and 0.76	None
AVF maturation [52]	AVF	492	Demographics and preoperative ultrasound	Maturation	Retrospective; internal	AUC 0.869; F1 0.883	None
Catheter conversion [50]	AVF	73,031	Demographic and clinical	Return to catheter within 1 year	Retrospective; internal	AUC 0.541–0.571	None
AVF thrombosis, interpretable RF [57]	AVF	974	Clinical and laboratory	Thrombosis	Retrospective; internal; no external validation	AUC 0.984	None
Optical monitoring, DeepVAQ [72]	NR	NR	Wearable or contact PPG	Access quality classification	Patient-level separation NR	Accuracy 92.13%; AUC 0.8605	None
Acoustic, lightweight CNN [83]	Mixed	119 patients, 3819 spectrograms	Blood-flow sound	Stenosis	Retrospective; sample-level analysis; no patient-level external validation	100% sample-level accuracy	None
Acoustic, longitudinal [88]	Arteriovenous access	84 patients, 437 weekly recordings	Mel spectrogram	Dysfunction requiring intervention within 10 days	Longitudinal; separate test set	AUC 0.7112	None
Acoustic, HVSI [89]	Mixed	202	Digital stethoscope sound index	Reduced access flow	Prospective, matched sampling (may overestimate screening performance)	AUC 0.90–0.94	None
Vibration, wireless thrill sensor [91]	AVG	9 (plus 5 canines, benchtop)	Dual MEMS accelerometers	Thrill intensity, lesion localization	Exploratory multi-platform; no diagnostic accuracy analysis	Not reported as AUC	None
Implantable pressure sensing, smart graft [97]	AVG	Phantom models only	Capacitive pressure, inductive coupling	Arterial and venous stenosis	Preclinical	NR	None

Abbreviations: AUC, area under the receiver operating characteristic curve; AVF, arteriovenous fistula; AVG, arteriovenous graft; CNN, convolutional neural network; DHRP, Dialysis Hospitalization Reduction Program; EHR, electronic health record; NR, not reported; PPG, photoplethysmography; RF, random forest; XGBoost, extreme gradient boosting. NR indicates that the item was not reported in the source publication. Reference standards, calibration, and sensitivity and specificity for each study are reported in Supplementary Table S1.

## Data Availability

No new data were created or analyzed in this study. Data sharing is not applicable to this article.

## Supplementary Materials

The following supporting information can be downloaded at: https://www.mdpi.com/article/10.3390/s26196089/s1, Table S1: Extended characteristics of the studies summarized in Table 1, including reference standard, unit of analysis, train/test separation strategy, calibration, sensitivity, specificity, study design, and funding; Table S2: Search strings used on 22 August 2026.
