# Supplementary material for: Artificial Intelligence and Sensing Technologies for Vascular Access in Hemodialysis: A Narrative Review
Source: Sensors (Basel). 2026 Sep 25;26(19):6089. doi: 10.3390/s26196089 (PMC13667549; doi:10.3390/s26196089)
Supplement: Supplementary file 1 [file sensors-26-06089-s001.zip › sensors-4562705-supplementary.pdf]

Table S1. Extended characteristics of the studies summarized in Table 1, including reference standard, unit of analysis, train/test separation strategy, calibration, sensitivity, specificity, study design, and funding.

Study	Reference standard	Unit of analysis	Train/test separation strategy	Calibration reported	Sensitivity	Specificity	Design (retrospective/prospective)	Funding and conflicts
[27]	Registry mortality	Patient	NR	Brier score 0.158	NR	NR	Retrospective	NR
[29]	Administrative hospitalization records	Clinic	Matched clinics	NR	NR	NR	Retrospective	NR
[40]	AVF failure definition per database	Patient	NR	Reported as good	NR	NR	Retrospective	NR
[54]	Recurrent intervention	Patient	Internal test set	NR	NR	NR	Prospective cohort	NR
[52]	Maturation criteria	Patient	NR	NR	NR	NR	Retrospective	NR
[50]	Catheter conversion	Patient	NR	NR	NR	NR	Retrospective	NR
[57]	Documented thrombosis	Patient	NR	NR	NR	NR	Retrospective	NR
[72]	Access quality label	Recording	NR	NR	NR	NR	NR	NR
[83]	Angiographic or clinical stenosis	Spectrogram	Sample-level split	NR	NR	NR	Retrospective	NR
[88]	Intervention within 10 days	Recording, longitudinal	Separate test set	NR	NR	NR	Prospective longitudinal	NR
[89]	Access flow threshold	Patient	Matched sampling	NR	NR	NR	Prospective	NR
[91]	Doppler ultrasound flow, DSA	Monitoring point	Not applicable	Not applicable	NR	NR	Exploratory	NR
[97]	Phantom stenosis	Device measurement	Not applicable	Not applicable	NR	NR	Preclinical	NR

Table S2. Search strings used used on 22 August 2026.

#	Database	Search string
1	PubMed	("hemodialysis"[tiab] OR "haemodialysis"[tiab] OR "renal dialysis"[MeSH]) AND ("vascular access"[tiab] OR "arteriovenous fistula"[tiab] OR "arteriovenous graft"[tiab] OR "Arteriovenous Shunt, Surgical"[MeSH]) AND ("artificial intelligence"[tiab] OR "machine learning"[tiab] OR "deep learning"[tiab] OR "neural network"[tiab] OR "prediction model"[tiab])
2	PubMed	("hemodialysis"[tiab] OR "haemodialysis"[tiab]) AND ("vascular access"[tiab] OR "arteriovenous fistula"[tiab] OR "arteriovenous graft"[tiab]) AND ("stenosis"[tiab] OR "thrombosis"[tiab] OR "maturation"[tiab] OR "failure"[tiab] OR "patency"[tiab]) AND ("machine learning"[tiab] OR "artificial intelligence"[tiab])
3	PubMed	("hemodialysis"[tiab] OR "haemodialysis"[tiab]) AND ("vascular access"[tiab] OR "arteriovenous fistula"[tiab] OR "arteriovenous graft"[tiab]) AND ("photoplethysmograph"[tiab] OR "acoustic"[tiab] OR "bruit"[tiab] OR "thrill"[tiab] OR "vibration"[tiab] OR "accelerometer"[tiab] OR "thermal sensor"[tiab] OR "pressure sensor"[tiab] OR "wearable"[tiab])
4	PubMed	("hemodialysis"[tiab] OR "haemodialysis"[tiab]) AND ("vascular access"[tiab] OR "arteriovenous fistula"[tiab] OR "arteriovenous graft"[tiab]) AND (robotic*[tiab] OR "near-infrared"[tiab] OR "augmented reality"[tiab] OR "virtual reality"[tiab] OR "ultrasound guidance"[tiab])
5	ClinicalTrials.gov	Condition/disease: hemodialysis OR haemodialysis; Other terms: (vascular access OR arteriovenous fistula OR arteriovenous graft) AND (artificial intelligence OR machine learning OR sensor OR wearable OR monitoring)
